# An Assessment of the Interoperability of Electronic Health Record Exchanges Among Hospitals and Clinics in Taiwan

**DOI:** 10.2196/12630

**Published:** 2019-03-28

**Authors:** Hsyien-Chia Wen, Wei-Pin Chang, Min-Huei Hsu, Cheng-Hsun Ho, Chi-Ming Chu

**Affiliations:** 1 School of Healthcare Administration College of Management Taipei Medical University Taipei Taiwan; 2 Graduate Institute of Bio-medical Informatics College of Medical Science and Technology Taipei Medical University Taipei Taiwan; 3 Graduate Institute of Information Management National Taipei University Taipei Taiwan; 4 Section of Biostatistics and Bioinformatics, Department of Epidemiology School of Public Health National Defense Medical Center Taipei Taiwan

**Keywords:** electronic health records, interoperability, data exchange, hospitals, clinics

## Abstract

**Background:**

The rapid aging of the Taiwanese population in recent years has led to high medical needs for the elderly and increasing medical costs. Integrating patient information through electronic health records (EHRs) to reduce unnecessary medications and tests and enhance the quality of care has currently become an important issue. Although electronic data interchanges among hospitals and clinics have been implemented for many years in Taiwan, the interoperability of EHRs has not adequately been assessed.

**Objective:**

The study aimed to analyze the efficiency of data exchanges and provide suggestions for future improvements.

**Methods:**

We obtained 30 months of uploaded and downloaded data of EHRs among hospitals and clinics. The research objects of this study comprised 19 medical centers, 57 regional hospitals, 95 district hospitals, and 5520 clinics. We examined 4 exchange EHR forms: laboratory test reports, medical images, discharge summaries, and outpatient medical records. We used MySQL (Oracle Corporation) software (to save our data) and phpMyAdmin, which is a Personal Home Page program, to manage the database and then analyzed the data using SPSS 19.0 statistical software.

**Results:**

The quarterly mean uploaded volume of EHRs among hospitals was 52,790,721 (SD 580,643). The quarterly mean downloaded volume of EHRs among hospitals and clinics was 650,323 (SD 215,099). The ratio of uploaded to downloaded EHRs was about 81:1. The total volume of EHRs was mainly downloaded by medical centers and clinics, which accounted for 53.82% (mean 318,717.80) and 45.41% (mean 269,082.10), respectively, and the statistical test was significant among different hospital accreditation levels (*F*_2_=7.63; *P*<.001). A comparison of EHR download volumes among the 6 National Health Insurance (NHI) branches showed that the central NHI branch downloaded 11,366,431 records (21.53%), which was the highest, and the eastern branch downloaded 1,615,391 records (3.06%), which was the lowest. The statistical test among the 6 NHI branches was significant (*F*_5_=8.82; *P*<.001). The download volumes of laboratory tests reports and outpatient medical records were 26,980,425 (50.3%) and 21,747,588 records (40.9%), respectively, and were much higher than medical images and discharge summaries. The statistical test was also significant (*F*=17.72; *P*<.001). Finally, the download time showed that the average for x-rays was 32.05 seconds, which was the longest, and was 9.92 seconds for electrocardiogram, which was the shortest, but there was no statistically significant difference among download times for various medical images.

**Conclusions:**

After years of operation, the Electronic Medical Record Exchange Center has achieved the initial goal of EHR interoperability, and data exchanges are running quite stably in Taiwan. However, the meaningful use of EHRs among hospitals and clinics still needs further encouragement and promotion. We suggest that the government’s leading role and collective collaboration with health care organizations are important for providing effective health information exchanges.

## Introduction

### Background

Many US studies have shown that electronic health records (EHRs) reduce repeated exams and medications, which can cause unnecessary costs, thereby improving patient safety and quality of care [1-8]. *The American Recovery and Reinvestment Act* of 2009 authorizes the Centers for Medicare and Medicaid Services (CMS) to award incentive payments to eligible professionals (EPs) and hospitals that demonstrate meaningful use of certified EHRs. The CMS EHR Incentive Program encourages EPs and hospitals to adopt EHRs and apply their meaningful use in 3 stages over 5 years. Clinically, the definition of meaningful use is using certified EHR technology to improve the quality, safety, and efficiency, which results in better clinical outcomes. Meaningful use sets specific objectives that EPs and hospitals must achieve to qualify for the CMS incentive programs. These specific objectives are divided into core and menu sets to check and pay those who meet these requirements, as detailed in the Clinical Quality Measure, such as electronically capturing health information in a standardized format, engaging in rigorous health information exchanges, and improving health outcomes [9].

Taiwan’s EHRs were initially developed by the National Health Informatics Project in 2004. The EHR exchange was implemented in 3 stages: stage 1 (2008-2011) began and promoted the EHR plan, stage 2 (2010-2012) accelerated EHR adoption in hospitals and clinics, and stage 3 (2013-2015) subsidized the interoperability and application of EHRs. According to statistics of the Electronic Medical Record (EMR) Exchange Center (EEC) in 2016, 411 of 496 hospitals (80.4%) and about 5244 of 9782 private clinics (53.6%) were certified as having interoperable EHRs [10].

**Figure 1:**
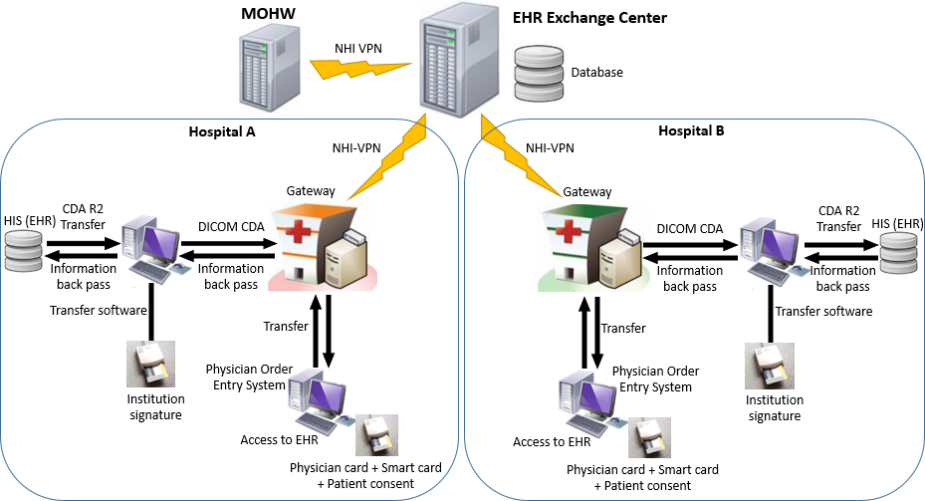
Configuration of the electronic health record data exchange system in Taiwanese hospitals. CDA: Clinical Document Architecture; DICOM: Digital Imaging and Communications in Medicine; EHR: electronic health record; HIS: hospital information system; MOHW: Ministry of Health and Welfare; NHI-VPN: National Health Insurance-Virtual Private Network.

**Figure 2:**
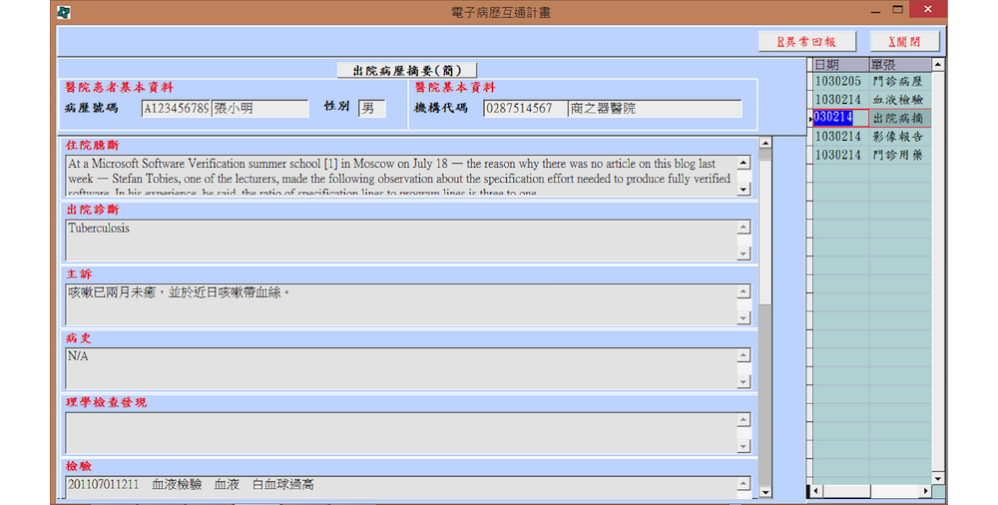
A retrieved discharge summary downloaded from the electronic health record exchange system.

On the basis of the configuration of the EHR data exchange system in Taiwanese hospitals (Figure 1), patients are allowed to use a National Health Insurance (NHI) integrated circuit (IC) card and can ask their physician to retrieve their medical information from hospitals they have previously visited. Patients have to sign a written consent form to authorize the physician before retrieving their medical records. The EEC functions only as an EHR index generation and search service platform for hospitals and clinics. Hospitals’ information systems are connected to the EEC through an EMR gateway. The hospital converts laboratory test reports, medical images, discharge summaries, and outpatient medical records in its EMR system into standardized files and saves them on the EMR gateway. The EEC generates an index of all XML files on EMR gateways of all hospitals and provides search and retrieval services for hospitals and clinics. In this system, Health Level 7 (HL7)/Clinical Document Architecture, Release 2 (CDA R2) standards are used to generate clinical documents and the Integrating the Healthcare Enterprise (IHE) Cross-enterprise Document Sharing (XDS) profile for the communication infrastructure. The EMR gateway receives clinical documents from the hospital information system, registers the metadata in the document registry (EEC), and stores them for 6 months. An example of a patient who asked a hospital to retrieve and download his discharge summary for receiving continuous care is illustrated in Figure 2.

Some large hospitals in Taiwan have met level-6 requirements of the EMR Adoption Model, which was built by the Healthcare Information and Management Systems Society [11,12]. Taking EMR information of Taichung Veterans General Hospital (TVGH) [12] as an example, almost 90% of medical records for outpatients, emergency visits, examinations, operations, and inpatients in this medical center were digitalized. As 80%-90% of patients registered before their visit to TVGH, drug duplications and interactions can automatically be detected by the exchange records through the EEC. Therefore, it can effectively alert physicians and prevent them from prescribing unnecessary medications and treatments.

In addition to continuously implementing paperless and interoperable medical records, Taiwan launched the Smart Healthcare plan in 2009, which uses information and communications technology (ICT) to provide a Pharma Cloud System [13] to reduce therapeutic duplications and enhance public medication safety. The Personal Health Bank [14,15], a set of personal health records applied in 2014, allows patients to access their health data for various health-enhancing applications. Under these innovative services, the delivery of integrated health care in Taiwan can become more convenient and effective.

### Objective

The study aimed to analyze the efficiency of data exchanges and provide suggestions for future improvements.

## Methods

Data of this study provided by the EEC of the Ministry of Health and Welfare consisted of 19 medical centers (>500 beds), 57 regional hospitals (>300 beds), 95 district hospitals (>100 beds), and 5520 clinics. The study period was from January 2015 to June 2017, for a total of 30 months. A total of 4 EHR exchange forms consisted of discharge summaries, outpatient records (including medication sheets), laboratory tests, and medical images. We used MYSQL software to save our data and used phpMyAdmin to manage them. Finally, we used SPSS 19.0 statistical software to analyze the variables, such as EHR exchange forms, accreditation level, geographic area, and time required for the EHRs to be downloaded.

## Results

### Descriptive Data of Uploaded and Downloaded Electronic Health Record Volumes

**Figure 3:**
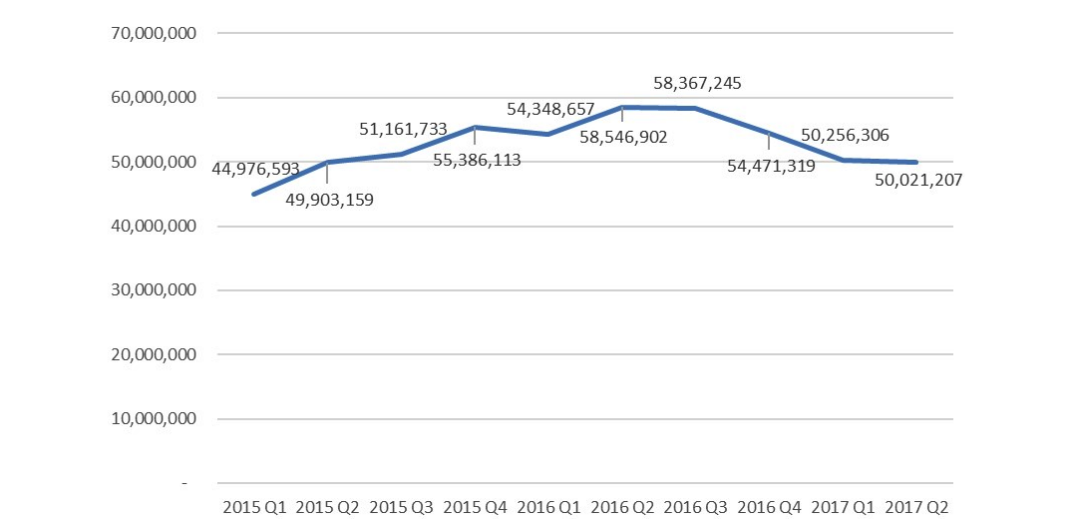
Electronic health record (EHR) volumes uploaded by hospitals from 2015 to 2017.

**Figure 4:**
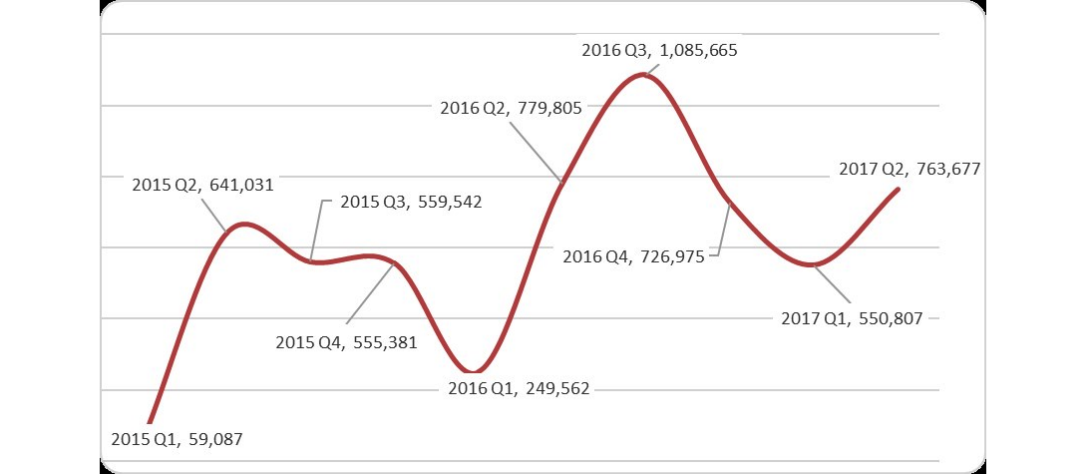
Electronic health record (EHR) volumes downloaded by hospitals and clinics from 2015 to 2017.

We used 3 months (1 quarter) as the analytical unit of EHR upload and download volumes, and the study period was from January 2015 to June 2017. The mean hospital uploaded EHR volume was 52,790,721, with an SD of 580,643, a maximum of 58,546,658, and a minimum of 45,549,300 records (Figure 3). The line in Figure 3 illustrates that the volume of uploads by hospitals tended to be stable. However, the volume of downloads by hospitals fluctuated (Figure 4). The mean hospital download volume was 650,323, with an SD of 215,099, a maximum of 1,085,665, and a minimum of 59,087 records. In the first quarter of each year, the download volume was significantly lower than the average because of the lunar new year vacation. The ratio of uploaded to downloaded EHRs was about 81:1.

### Variables Affecting the Uploaded Volume of Electronic Health Records

Hospital upload and download volumes of EHRs were analyzed by hospital accreditation level, NHI branch, and EHR format, as shown in Table 1. First, average volumes of uploaded EHRs ranked by hospital accreditation of medical centers (>500 beds) with 200,927,617, regional hospitals (>300 beds) with 223,199,389, and district hospital (>100 beds) with 10,378,021 records, which reached statistical significance (*F*=154.81; *P*<.001). A comparison of the 6 NHI branches representing 6 different geographical regions of Taiwan showed that the Taipei branch had the highest average volume with 18,031,142, whereas the eastern branch had the lowest with 1,615,391 records, and the statistical test was significant (*F*=360.52; *P*<.001). Moreover, the 4 EHR exchange forms indicated that the highest volume was for laboratory tests with 26,980,425.5 records. Discharge summaries were the lowest with 675,093.5 records, and this was significant according to the statistical test (*F*=887.23; *P*<.001).

**Table 1:** Uploaded and downloaded volumes of electronic health records for different accreditation levels, National Health Insurance branches, and electronic health record forms.

Variable and category			Mean (SD)	*F* value (degrees of freedom)	*P* value	Scheffe test
**Uploaded EHRs^a^**						
	**Accreditation level**					
		Medical centers (A); District hospitals (B); Regional hospitals (C)	20,092,761.7 (1,169,657.3); 22,319,938.9 (2,300,920.9); 10,378,021.1 (1,073,403.2)	154.8 (2)	<.001	A>C; B>A, C
	**NHI^b^** **branch**					
		Taipei (A); Northern (B); Central (C); Southern (D); Kaoping (E); Eastern (F)	18,031,142.3 (1,771,219); 6,444,815.4 (636,379.5); 11,366,431.6 (60,1067.2); 7,453,093.0 (708,056.1); 7,898,723.0 (790,655.7); 1,615,391.7 (125,642.2)	360.5 (5)	<.001	A>B, C, D, E, F; B>F; C>B, D, E, F; D>B, F; E>B, D
	**EHR form**					
		Laboratory tests (A); Discharge summaries (B); Medical images (C); Outpatient records (D)	26,980,425.5 (2,053,721.2); 675,093.5 (81,408.80); 3,406,489.6 (302,759.2); 21,747,588.4 (1,853,264.3)	887.2 (3)	<.001	A>B, C, D; C>B; D>B, C
**Downloaded EHRs**						
	**Accreditation level**					
		Medical centers (A); Regional hospitals (B); District hospitals (C); Clinics (D)	318,717.8 (332,577.7); 2,523.4 (1623.5); 2,286.3 (996.2); 269,082.1 (199,866.6)	7.63 (3)	<.001	A>B, C, D; B>C; D>B, C
	**NHI branch**					
		Taipei (A); Northern (B); Central (C); Southern (D); Kaoping (E); Eastern (F)	130,172.5 (62,431.2); 69,089.4 (37,708.3); 305,230.0 (272,800.0); 58,500.3 (44,413.6); 15,463.7 (12,255.4); 14,173.4 (8116.1)	8.82 (5)	<.001	A>B, D, E, F; B>D, E, F; C>A, B, D, E, F; D>E, F, F
	**EHR form**					
		Laboratory tests (A); Discharge summaries (B); Medical images (C); Outpatient records (D)	242,572.3 (168,448.7); 10,081.2 (4778.7); 42,038.8 (19,924.8); 297,937.0 (132,678.9)	17.72 (3)	<.001	A>B, C; C>B; D>A, B, C

^a^EHRs: electronic health records.

^b^NHI: National Health Insurance.

### Variables Affecting the Downloaded Volumes of Electronic Health Records

Table 1 shows that the highest average volume was 318,717.8 downloads by medical centers, followed by clinics with 269,082.1, and the statistical test among hospital accreditation levels was significant (*F*=7.63; *P*<.001). In a comparison of EHR downloaded volumes among the 6 NHI branches, the central NHI branch had the highest volume with 305,230 downloads, and the eastern branch had the lowest volume with 14,173.4 downloads, and it was also statistically significant among the 6 NHI branches (*F*=8.82; *P*<.001). In addition, among downloaded volumes of the 4 EHR forms, outpatient medical records was the highest with 297,937.0 downloads, followed by laboratory tests with 242,572.3, which were both much higher than those for medical images and discharge summaries, and the statistical test was also significant (*F*=17.72; *P*<.001).

Furthermore, we focused on the average download times of the 6 medical images. Table 2 shows that x-rays took the longest at 32.05 seconds, and electrocardiogram (EKG) took 9.92 seconds, which was the shortest, but there was no statistically significant difference. Finally, when each district was compared with the other 5 districts according to the geographical location in Taiwan, all the highest download volumes occurred within that district as illustrated in Table 3. This indicates that patients tended to obtain treatments within their daily living sphere instead of crossing into a different area, possibly to avoid the extra cost and inconvenience of transportation.

**Table 2:** Required download times for different medical images. *F* value (degrees of freedom)=0.29 (5); *P*=.91.

Medical images	Mean (SD)
Ultrasound	21.3 (10.4)
X-ray	32.1 (42.8)
Computed tomography	20.3 (7.9)
Magnetic resonance image	31.3 (35.4)
Endoscopy	23.6 (13.2)
Electrocardiogram	9.9 (9.9)

**Table 3:** Download volumes of electronic health records among six different districts in Taiwan.

Downloaded by	Districts					
	Taipei	Northern	Central	Southern	Kaoping	Eastern
Taipei	1,093,087	169,168	23,674	18,851	9793	6428
Northern	95,138	562,343	31,288	4856	4248	2509
Central	12,773	9415	787,002	23,384	4865	889
Southern	8154	4188	19,085	540,814	15,266	549
Kaoping	1503	1861	1194	5329	146,179	278
Eastern	4671	1640	655	353	1025	133,664

## Discussion

### Upload Volumes of Electronic Health Records Among Hospitals Tended to Be Steady

The vision of Taiwan’s ECC is that hospitals’ and clinics’ physicians can view patients’ most recent 6 months of medical records with the patient’s consent and the physician’s authorization using the patient’s NHI IC card and the physician’s professional IC card. Hospitals’ upload volumes of EHRs were about 4.5 million to 60 million quarterly (average 52,790,721) during the study period from January 2015 to June 2017. In the beginning, we estimated that upload volumes would slowly decrease as the government’s subsidy ended. However, hospital upload volumes of EHRs were quite stable. A possible reason is that the EHR project management office [16], an official EHR exchange standards task force in Taiwan in charge of EHR training, education, demonstration, awards, and subsidies for hospitals and clinics, continued monitoring hospitals’ compliance by publishing their weekly upload volumes. Therefore, hospitals had to conduct EHR uploads as a daily operation, and many hospitals used the application programming interface (API) [12] to automatically meet the requirements of the EEC. Figure 3 illustrates that hospital uploads were well maintained and tended to be steady.

Moreover, based on the service capacity and investment of hospital information systems, it is understandable that the total upload volumes of EHRs by medical centers (>500 beds) and regional hospitals (>300 beds) were significantly higher than those by district hospitals (>100 beds).

### Download Volumes of Electronic Health Records Among Hospitals and Clinics Fluctuated

The average quarterly volume of EHR downloads by hospitals and clinics was 592,629 records. Compared with the upload volume listed above, the ratio was about 81:1. In other words, when hospitals uploaded 81 EHRs, only 1 was downloaded by physicians for medical purposes. Under the current policy of the NHI, although the integrated medical care and referral system was strongly promoted and encouraged, the download volumes of EHRs among hospitals and clinics fluctuated. On the basis of physicians’ practice behaviors and hospital accreditation, Table 1 shows that medical centers (>500 beds) and clinics downloaded 318,717.8 and 269,082 records, respectively, which accounted for more than 95% of the total download volume. This means that more efforts are needed to implement 2-way referrals among hospitals and clinics for patients’ continuous care. Moreover, Table 3 illustrates that each district’s internal download volume was significantly higher than that of the other 5 different districts. We believe that patients were constrained by time and costs, so seeking cross-regional medical treatment is rare in Taiwan, and this also proved a resident life circle exists in local people.

In Taiwan, in addition to EHR exchanges provided by the EEC, the NHI administration also launched a Pharma Cloud system in 2015 [17]. Physicians in hospitals and clinics were requested to inquire into patients’ medications during previous visits throughout this system. Under the single-payer NHI system, hospitals and clinics have to comply with regulations of the NHI administration, or otherwise, their reimbursements will be denied. However, download volumes of EHRs did not decline because of the NHI administration’s service. On the contrary, as Figure 4 illustrates, volumes significantly increased in 2016 because of the API for the EEC. It facilitated hospitals automatically downloading EHRs a day before a patient’s visit. Especially in the third quarter of 2016 (Figure 4), the download volume surged because medical centers put a lot of effort into EHR exchanges to meet the requirements of hospital accreditation.

We believe that effective health information exchange can avoid repeated examinations and medications [18,19] and reduce unnecessary medical expenses [6,20]. Thus, the CMS of the United States requires health care providers who accept government subsidies to achieve the criteria of meaningful use [9]. Although the volume of EHRs exchanged by hospitals and clinics gradually increased in Taiwan, whether it really contributes to the continuous care of patients is still unclear and worthy of further study.

### Download Times of the 4 Electronic Health Record Forms Required by Hospitals and Clinics Did Not Significantly Differ

Table 2 illustrates that download times for ultrasound, EKGs, computed tomography, magnetic resonance imaging, and endoscopic images, and x-rays were between 9.92 and 32.05 seconds. When hospitals and clinics downloaded different types of medical images, the required times did not significantly differ (*P*>.914). However, when we interviewed some physicians, they complained that the waiting times for downloads were too long, especially for x-rays, and it definitely affected their intention to use this service. The gap between our data and physicians’ experiences may come from the time measurement. We counted the time from when the EEC received a request from a hospital to when the gateway at the hospital received the downloaded medical image. Due to high outpatient service volumes and a variety of physician practice styles, it was difficult for us to detect how much time elapsed from the hospital gateway to the physician’s terminal when these medical images were being checked. Moreover, in some hospitals, the computing priority of the information system may have affected download speeds, so physicians were unable to promptly review downloaded images.

### Building Up an Integrated Cross-Hospital Health Care Model

Data exchanges among health organizations are based on interoperable EHRs. The services provided by EEC met the requirements of functional and semantic interoperability. We adopted the HL7/CDA R2 and Digital Imaging and Communications in Medicine standards to generate clinical documents and IHE XDS profile for the communication infrastructure. Therefore, a physician can read laboratory test reports, medical images, discharge summaries, and outpatient medical records through these interoperability standards by EHR exchanges. Taiwan has invested more than 10 years in building up the EHR infrastructure and legal system [21]. The final goal of the government is to provide continuous care for patients and achieve cross-hospital integrated health care. Many similar examples in the United States and Europe have actively implemented cross-hospital integrated health care. Regional Health Information Exchange Organizations in the United States, which are in charge of cross-regional data exchanges, have focused on the meaningful use in various health care settings. Kaiser Permanente Health Connect [22] provides continuous care to 1.1 million insured persons through EHRs from its 38 hospitals and 650 clinics and gives patients the latest medical information. SmartCare [23] in Europe has developed a standard interoperable platform to share data with 23 regional stakeholders to provide integrated health care services. A LinkCare project called NEXES, an integrated health care shared knowledge community, supports healthy and independent living by chronically ill patients and the elderly by health care professionals. It is an ICT-enabled integrated care program in large-scale trials (5200 patients), targeting prevalent chronic conditions (mainly chronic obstructive pulmonary disease, chronic heart failure, and diabetes) and is being run in Barcelona and Alicante, Spain, and Athens, Greece, in Europe [24]. We believe that the interoperability and connectivity of EHRs are not only the future of health care but they are also big challenges around the globe. Thus, the government has to play a leading role in defining policies and offering incentives to encourage health care organizations to engage in cross-hospital integrated health care.

### Limitations

Due to high outpatient service volumes and a variety of physician practice styles, we could not detect how much time it took from hospital gateways to physicians’ terminals when medical data were being checked. We suggest that future studies measure the time physicians actually spend on data review except on data only from the ECC.

### Conclusions

After years of operation, volumes of EHRs uploaded to the EEC by hospitals were stable but download volumes of EHRs fluctuated. The primary goal of the EEC for promoting cross-hospital data exchanges was achieved. However, the meaningful use of EHRs among hospitals and clinics needs further encouragement and promotion for reducing unnecessary medications and examinations and enhance the quality of care. We suggest that the government’s leading role and collective collaboration with health care organizations are important keys to providing effective health information exchanges.

## Abbreviations

API: application programming interface
CDA R2: Clinical Document Architecture, Release 2
CMS: Centers for Medicare and Medicaid Services
EEC: Electronic Medical Record Exchange Center
EHR: electronic health record
EKG: electrocardiogram
EMR: electronic medical record
EP: eligible professional
HL7: Health Level 7
IC: integrated circuit
ICT: information and communications technology
IHE: Integrating the Healthcare Enterprise
NHI: National Health Insurance
TVGH: Taichung Veterans General Hospital
XDS: Cross-enterprise Document Sharing
